# Multi-copper ferroxidase deficiency leads to iron accumulation and oxidative damage in astrocytes and oligodendrocytes

**DOI:** 10.1038/s41598-019-46019-9

**Published:** 2019-07-01

**Authors:** Zheng Chen, Ruiwei Jiang, Mengxia Chen, Jiashuo Zheng, Min Chen, Nady Braidy, Shunli Liu, Guohao Liu, Zaitunamu Maimaitiming, Tianqi Shen, Joshua L. Dunaief, Christopher D. Vulpe, Gregory J. Anderson, Huijun Chen

**Affiliations:** 1 Jiangsu Key Laboratory of Molecular Medicine,, Medical School of Nanjing University, Nanjing, China; 2 Institute of Virology, Wenzhou University, Wenzhou, China; 3 School of Medicine, Xiamen University, Xiamen, China; 4 Centre for Healthy Brain Ageing, UNSW School of Psychiatry, NPI, Euroa Centre, Prince of Wales Hospital, Barker Street, Randwick, Sydney, NSW Australia; 5 FM Kirby Center for Molecular Ophthalmology, Scheie Eye Institute, University of Pennsylvania, Philadelphia, PA USA; 6 Center for Environmental and Human Toxicology, Department of Physiological Sciences, University of Florida, Gainesville, FL USA; 7 QIMR Berghofer Medical Research Institute, Brisbane, Queensland Australia

**Keywords:** Glial biology, Metabolism

## Abstract

Accumulation of iron has been associated with the pathobiology of various disorders of the central nervous system. Our previous work has shown that hephaestin (*Heph*) and ceruloplasmin (*Cp*) double knockout (KO) mice induced iron accumulation in multiple brain regions and that this was paralleled by increased oxidative damage and deficits in cognition and memory. In this study, we enriched astrocytes and oligodendrocytes from the cerebral cortex of neonatal wild-type (WT), *Heph* KO and *Cp* KO mice. We demonstrated that *Heph* is highly expressed in oligodendrocytes, while *Cp* is mainly expressed in astrocytes. Iron efflux was impaired in *Cp* KO astrocytes and *Heph* KO oligodendrocytes and was associated with increased oxidative stress. The expression of *Heph*, *Cp*, and other iron-related genes was examined in astrocytes and oligodendrocytes both with and without iron treatment. Interestingly, we found that the expression of the mRNA encoding ferroportin 1, a transmembrane protein that cooperates with CP and HEPH to export iron from cells, was positively correlated with *Cp* expression in astrocytes, and with *Heph* expression in oligodendrocytes. Our findings collectively demonstrate that HEPH and CP are important for the prevention of glial iron accumulation and thus may be protective against oxidative damage.

## Introduction

Iron is a critical co-factor in a wide range of proteins, including many enzymes and proteins involved in oxygen transport^1^, by virtue of its capacity to shuttle between the ferrous (Fe^2+^) and ferric (Fe^3+^) forms. It is well established that free ferrous iron can facilitate the generation of toxic oxygen radicals, thus its levels are carefully controlled within both individual cells and tissues as a whole^2^. Multi-copper ferroxidases (MCFs) convert Fe^2+^ to Fe^3+^, and in doing so promote iron efflux from cells through the iron transporter, ferroportin 1 (FPN1)^3^. Three MCFs have been characterized in mammalian cells - ceruloplasmin (CP), hephaestin (HEPH) and zyklopen (ZP)^4–6^. In this study we have focused on CP and HEPH as both have been shown to play important roles in brain iron metabolism. Our understanding of iron homeostasis and transport in the brain is evolving steadily and this area has been well reviewed eleswhere^7,8^. In this study, we examined the expression of key components of cellular iron metabolism which are regulated by iron levels, including FPN1, transferrin receptor 1 (TFRC), divalent metal-ion transporter 1 (DMT1), and ferritin light (L-ferritin) and heavy chain (H-ferritin) to provide insight into the mechanisms by which iron homeostasis is regulated in astrocytes and oligodendrocytes.

Iron is a trace element that is essential for the central nervous system (CNS). Increased iron accumulation in multiple brain regions has been associated with the pathogenesis of several neurodegenerative diseases, including Alzheimer’s disease, Parkinson’s disease and multiple sclerosis^9,10^. Ferrous iron is associated with the pathogenesis of neurodegenerative diseases as it can enhance reactive oxygen species generation via the Fenton reaction^2,11^. It was previously thought that cytotoxicity arose solely within vulnerable neurons, triggering degeneration and neuronal death^12^. However, this view is changing with the growing understanding that astrocytes are associated with disease development and/or progression^12,13^. For example, mutations in the *CP* gene which impair the production of the protein (aceruloplasminemia) have been associated with neurological symptoms which reflects iron accumulation within the astrocytes^14^.

Impaired *Heph* or *Cp* function induces cell-specific iron deposition and neurological defects^15–17^. Several glial cell types can influence iron metabolism within the CNS^14^, including oligodendrocytes, astrocytes and microglia. CP is expressed as a membrane-bound GPI-linked protein on the astrocyte surface^18^, and consequently, individuals with aceruloplasminemia display significant iron deposition in astrocytes and often develop dementia and ataxia^19^. Consistent with this, iron accumulation was observed in the astrocytes of 24-month-old mice lacking CP^15^. Furthermore, sex-linked anemia (*sla*) mice have an in-frame deletion within *Heph* that leads to a significant reduction in ferroxidase activity^6,20^. These animals demonstrate increased iron content in gray matter oligodendrocytes by 2–3 months of age, although no significant differences are observed in the white matter, and motor deficits by 6 months of age^17^.

Our previous work has demonstrated that deletion of both *Heph* and *Cp* in mice leads to iron accumulation in various brain regions, with associated oxidative damage and impaired memory and learning^21–23^. However, the roles of these MCFs in specific glial cells are incompletely understood. We therefore enriched astrocytes and oligodendrocytes from the cerebral cortex of neonatal wild-type (WT), *Heph* KO and *Cp* KO mice and used them to study the effect of MCFs ablation on glial iron metabolism and the expression of iron related-genes.

## Results

### *Heph* and *Cp* gene expression in astrocytes and oligodendrocytes

**Figure 1:**
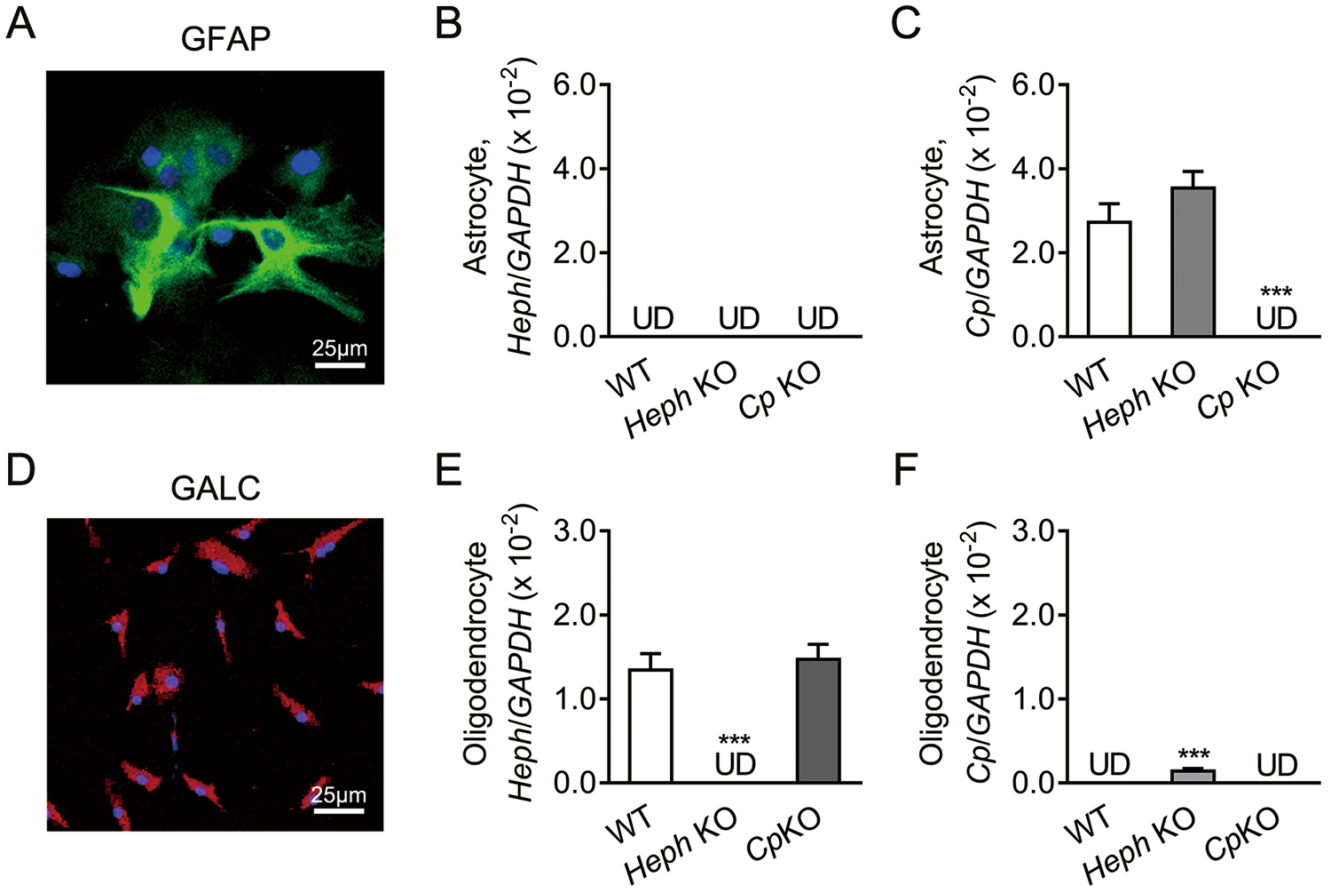
*Heph* and *Cp* gene expression in astrocytes and oligodendrocytes. (**A**) Representative images of WT astrocytes stained with the astrocyte marker GFAP (green) and DAPI (blue) to show the nuclei. (**B**,**C**) *Heph* (**B**) and *Cp* (**C**) mRNA levels in WT, *Heph* KO, and *Cp* KO astrocytes. (**D**) Representative images of WT oligodendrocytes stained with the oligodendrocyte marker GalC (red) and DAPI (blue) to show the nuclei. (**E**,**F**) *Heph* (**E**) and *Cp* (**F**) mRNA levels in WT, *Heph* KO, and *Cp* KO oligodendrocytes. Data are representative of at least three independent experiments. UD: undetectable; values are presented as the means ± SEMs; ***P < 0.001, different from WT, as determined by unpaired Student’s *t*-test.

Enriched populations of astrocytes and oligodendrocytes were isolated from the cerebral cortexes of neonatal WT, *Heph* KO, and *Cp* KO mice. Briefly, the cerebral cortex was triturated and passed through a 70 µm cell strainer. The filtered cells were maintained in DMEM supplemented with 10% FBS. After 10–12 days, confluent cultures were shaken overnight, and the floating cells were discarded to obtain a highly enriched monolayer of astrocytes. For oligodendrocytes, mechanically separated cerebral cortex cells were cultured in neurosphere growth medium (NPM). After 10 days, the NPM was gradually changed to oligosphere medium by replacing one-fourth of the former medium with the latter every other day. After 10 days in culture, the oligospheres were resuspended and maintained in OPC medium for OPC proliferation, or in oligodendrocyte differentiation medium for differentiation (see Materials and methods for details). Staining for the astrocyte marker GFAP (Fig. 1A) or the oligodendrocyte marker GalC (Fig. 1D) showed that the purities of the relevant isolated cell populations was 95% and 85% respectively. No *Heph* mRNA was detectable in astrocytes from any of the mouse strains (Fig. 1B), while *Cp* mRNA was abundant in WT and *Heph* KO astrocytes (Fig. 1C). In contrast, *Heph* mRNA was abundant in WT and *Cp* KO oligodendrocytes (Fig. 1E), while *Cp* mRNA was detected only at low levels in the same cells (Fig. 1F). Of note, we observed significantly higher *Cp* mRNA expression in *Heph* KO oligodendrocytes than in WT oligodendrocytes (*P* < 0.001), despite the low absolute value.

### Changes in iron efflux and intracellular oxidative stress in *Cp* KO astrocytes and *Heph* KO oligodendrocytes

**Figure 2:**
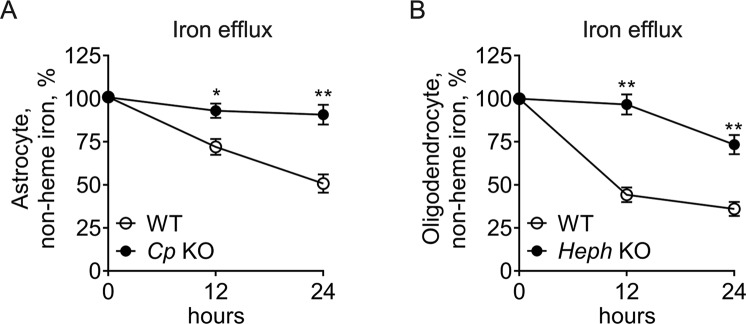
Iron efflux from *Cp* KO astrocytes and *Heph* KO oligodendrocytes. Non-heme iron concentrations in WT and *Cp* KO astrocytes (**A**), and WT and *Heph* KO oligodendrocytes (**B**) at 0, 12, 24 hours after the cells had been treated with iron then transferred to fresh medium without added iron. Iron within the cells at the 0 hour time point was set to 100%. Data are representative of at least three independent experiments. Values are presented as the means ± SEMs; *P < 0.05, **P < 0.01, different from WT, as determined by unpaired Student’s *t*-test.

**Figure 3:**
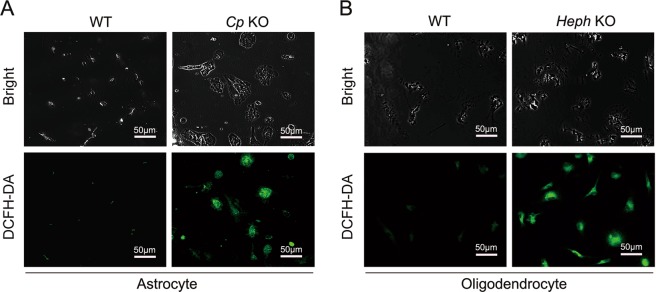
Intracellular oxidative stress in *Cp* KO astrocytes and *Heph* KO oligodendrocytes after iron treatment. WT and *Cp* KO astrocytes (**A**), and WT and *Heph* KO oligodendrocytes (**B**) were loaded with iron for 24 hours, then incubated for a further 24 hours in medium without iron. The cells were then stained with DCFH-DA to detect reactive oxygen species. Representative brightfield (top) and fluorescent (bottom) images are shown. Data are representative of at least three independent experiments.

Iron efflux was examined in WT and *Cp* KO astrocytes, or WT and *Heph* KO oligodendrocytes (Fig. 2A,B). The amount of iron in WT astrocytes pre-loaded with iron declined by approximately 30% over 12 hours after re-incubation of the cells in serum free medium without added iron, but under the same conditions only 7% of the iron was lost from *Cp* KO astrocytes (*P* < 0.05 compared with WT cells) indicating severely compromised iron efflux (Fig. 2A). At 24 hours after iron removal, the disparity between *Cp* KO and WT astrocytes was even more significant (*P* < 0.01). Relative to astrocytes, WT oligodendrocytes released a much greater proportion of their iron, with only 45% remaining 12 hours after iron removal (Fig. 2B). *Heph* KO oligodendrocytes showed a marked impairment in iron efflux relative to WT oligodendrocytes (*P* < 0.01) at both 12 and 24 hours after transfer to a low iron medium. Some efflux of iron (approximately 25%) was noted in *Heph* KO oligodendrocytes 24 hours after iron removal, but the difference between *Heph* KO and WT oligodendrocytes remained highly significant (*P* < 0.01). Using DCFH-DA staining, we reported that *Cp* KO astrocytes and *Heph* KO oligodendrocytes that retained iron after 24 hours in low iron medium had enhanced intracellular oxidative stress relative to their corresponding WT controls (Fig. 3A,B). These results suggest that impaired iron efflux contributed to oxidative stress in *Cp* KO astrocytes and *Heph* KO oligodendrocytes.

### Expression of some iron homeostasis genes in WT, *Heph* KO, and *Cp* KO astrocytes with or without iron treatment

**Figure 4:**
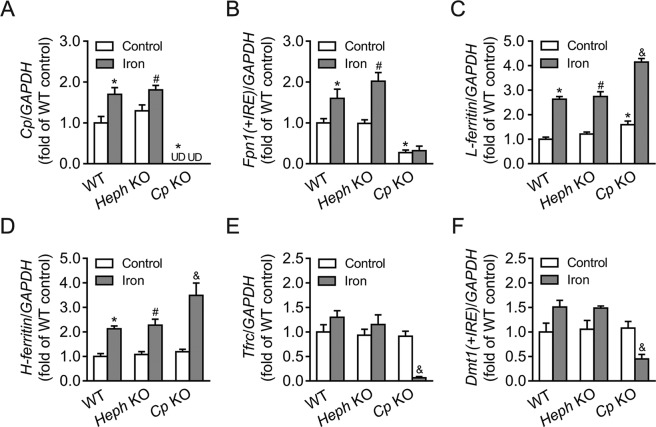
Expression of iron-related genes in WT, *Heph* KO, and *Cp* KO astrocytes with or without iron treatment. *Cp* (**A**), *Fpn1(*+*IRE)* (**B**), *L-ferritin* (**C**), *H-ferritin* (**D**), *Tfrc* (**E**) and *Dmt1(*+*IRE)* (**F**) mRNA levels were evaluated by quantitative PCR in WT, *Heph* KO, and *Cp* KO astrocytes treated with medium alone or with 40 μM iron. Data are representative of at least three independent experiments. UD: undetectable; values are presented as the means ± SEM; *P < 0.05, different from WT control; ^#^*P* < 0.05, different from *Heph* KO control; ^&^*P* < 0.05, different from *Cp* KO control, as determined by unpaired Student’s *t*-test.

The expression of *Cp*, *Fpn1(*+*IRE)*, *L-ferritin*, *H-ferritin*, *Tfrc*, and *Dmt1(*+*IRE)* mRNAs in WT, *Heph* KO, and *Cp* KO astrocytes was quantified by qRT-PCR (Fig. 4A–F). In the absence of iron treatment, *Cp* KO astrocytes displayed a significantly lower level of *Fpn1(*+*IRE)* mRNA (*P* < 0.05) and a significantly higher level of *L-ferritin* mRNA (*P* < 0.05), than WT controls. However, no significant differences were observed in *H-ferritin*, *Tfrc*, or *Dmt1(*+*IRE)* mRNA levels. No significant changes in the expression of any of the genes were observed between *Heph* KO and WT astrocytes in the absence of iron treatment. When treated with iron, *Cp* and *Fpn1(*+*IRE)* mRNA levels were significantly increased in WT and *Heph* KO astrocytes (both *P* < 0.05) relative to cells not treated with iron, but no difference was observed in *Cp* KO astrocytes where *Fpn1(*+*IRE)* mRNA expression was low. Both *L-ferritin* and *H-ferritin* mRNA levels were substantially increased in all three groups after iron treatment (all *P* < 0.05 vs cells not treated with iron). No significant differences were noted in *Tfrc* or *Dmt1(*+*IRE)* mRNA levels in *Heph* KO compared to WT astrocytes either before or after iron treatment, but their mRNA expression was significantly decreased in *Cp* KO astrocytes following the addition of iron (*P* < 0.05). These results suggest that ablation of *Cp*, but not *Heph*, had a major impact on the expression of genes encoding proteins involved in iron storage (*L-ferritin*, *H-ferritin*) and iron import (*Tfrc*, *Dmt1(*+*IRE)*) in astrocytes, especially after iron treatment. In contrast, the gene encoding the iron exporter FPN1 (*Fpn1(*+*IRE)*) remained at a low level in *Cp* KO astrocytes regardless of the iron treatment. These data support the notion that CP facilitates iron release from astrocytes in conjunction with FPN1, and that *Cp* KO induced iron overload in astrocytes.

### Expression of iron metabolism-related genes in WT, *Heph* KO, and*Cp* KO oligodendrocytes with or without iron treatment

**Figure 5:**
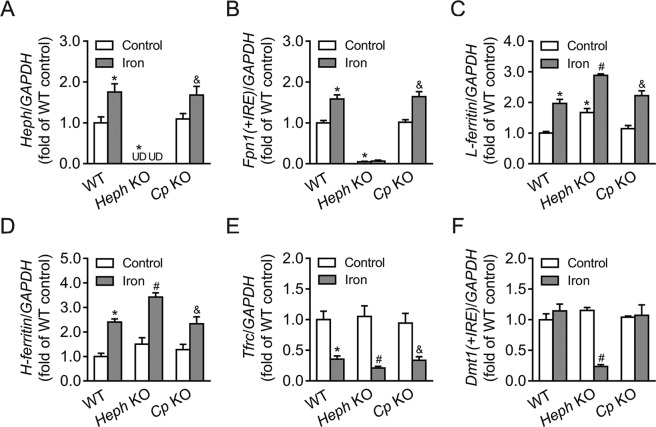
Expression of iron-related genes in WT, *Heph* KO, and *Cp* KO oligodendrocytes with or without iron treatment. *Cp* (**A**), *Fpn1(*+*IRE)* (**B**), *L-ferritin* (**C**), *H-ferritin* (**D**), *Tfrc* (**E**) and *Dmt1(*+*IRE)* (**F**) mRNA levels were evaluated by quantitative PCR in WT, *Heph* KO, and *Cp* KO oligodendrocytes treated with medium alone or with 40 μM iron. Data are representative of at least three independent experiments. UD: undetectable; values are presented as the means ± SEMs; *P < 0.05, different from WT control; ^#^*P* < 0.05, different from *Heph* KO control; ^&^*P* < 0.05, different from *Cp* KO control, as determined by unpaired Student’s *t*-test.

The expression of *Heph*, *Fpn1(*+*IRE)*, *L-ferritin*, *H-ferritin*, *Tfrc*, and *Dmt1(*+*IRE)* mRNAs in WT, *Heph* KO, and *Cp* KO oligodendrocytes was quantified by qRT-PCR (Fig. 5A–F). Consistent with the *Cp* KO astrocytes, *Heph* KO oligodendrocytes in the absence of iron treatment displayed a significantly lower level of *Fpn1(*+*IRE)* mRNA (*P* < 0.05) and a significantly higher level of *L-ferritin* mRNA (*P* < 0.05), compared with WT controls. No significant differences were observed in *H-ferritin*, *Tfrc*, or *Dmt1(*+*IRE)* expression, and no significant differences in the expression of any of the genes tested were observed between *Cp* KO and WT oligodendrocytes. *Heph* and *Fpn1(*+*IRE)* mRNA levels were increased in WT and *Cp* KO oligodendrocytes (both *P* < 0.05) following iron treatment, relative to untreated cells, but in *Heph* KO oligodendrocytes *Fpn1(*+*IRE)* mRNA expression was very low and showed little response to iron. *L-ferritin* and *H-ferritin* mRNA levels were significantly increased, and *Tfrc* mRNA levels were substantially decreased, in oligodendrocytes from all three mouse strains after iron treatment (all *P* < 0.05). No significant differences were noted in *Dmt1(*+*IRE)* mRNA levels in *Cp* KO or WT oligodendrocytes after iron treatment, but levels were significantly decreased in *Heph* KO oligodendrocytes under these conditions (*P* < 0.05). These results suggest that ablation of *Heph*, but not *Cp*, has a profound effect on the expression of genes encoding proteins associated with iron storage (*L-ferritin*, *H-ferritin*) and import (*Tfrc*, *Dmt1(*+*IRE)*) in oligodendrocytes, especially following the addition of iron. Similar to *Cp* KO astrocytes, *Fpn1(*+*IRE)* was expressed at a low level in *Heph* KO oligodendrocytes regardless of the iron treatment. These data support the notion that *Heph* facilitates iron release from oligodendrocytes in conjunction with FPN1, and that *Heph* KO induced iron overload in oligodendrocytes.

### Expression of iron metabolism-related genes in WT, *Heph* KO, and *Cp* KO oligodendrocyte precursor cells

**Figure 6:**
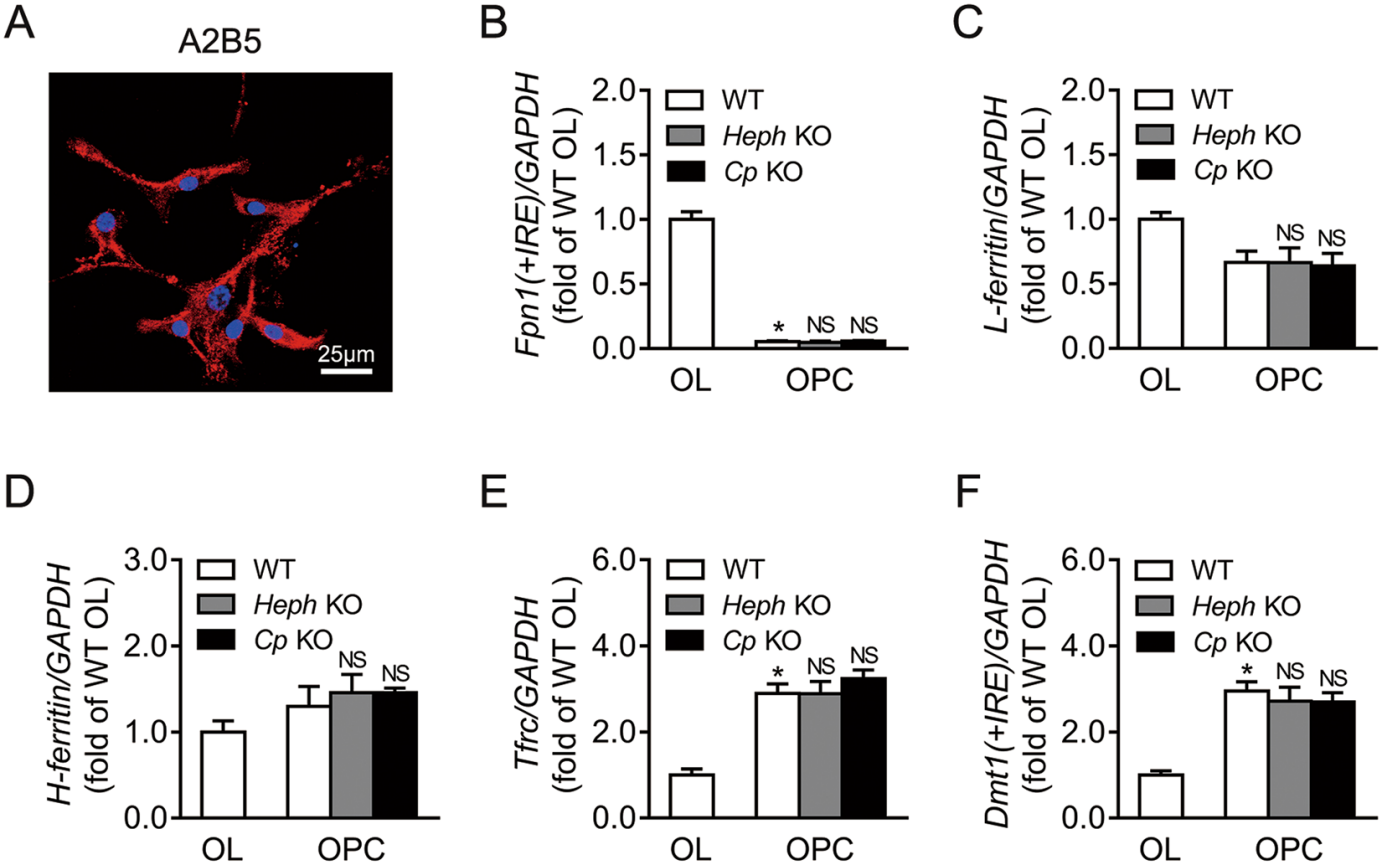
Expression of iron-related genes in WT, *Heph* KO, and *Cp* KO OPCs. (**A**) Representative image of WT OPCs stained with the OPC marker A2B5 (red) and DAPI (blue) to show the nuclei. (**B–F**) *Fpn1(*+*IRE)* (**B**), *L-ferritin* (**C**), *H-ferritin* (**D**), *Tfrc* (**E**) and *Dmt1(*+*IRE)* (F) mRNA levels in WT, *Heph* KO, and *Cp* KO OPCs. Expression in WT OLs is also shown. Data are representative of at least three independent experiments. OPC: oligodendrocyte precursor cell, OL: oligodendrocyte; UD: undetectable; NS: not significant vs WT OPCs; values are presented as the means ± SEMs; *P < 0.05, different from WT OLs, as determined by unpaired Student’s *t*-test.

Like mature glial cells, OPCs also require iron for their function, and there is evidence to suggest that this differs in at least some respects from that of mature oligodendrocytes^17,24^. Thus we also investigated the expression of iron-related genes in OPCs. WT OPCs were stained with the OPC marker A2B5 (Fig. 6A) and shown to have a purity of 80%. Neither *Heph* nor *Cp* mRNA was detected in any of the OPCs isolated (data not shown). The expression of *Fpn1(*+*IRE)*, *L-ferritin*, *H-ferritin*, *Tfrc*, and *Dmt1(*+*IRE)* mRNA in WT, *Heph* KO, and *Cp* KO OPCs was quantified by qRT-PCR (Fig. 6B–F). There was little effect of *Heph* or *Cp* ablation on the expression of iron-related genes in OPCs. WT OPCs displayed a significantly lower level of *Fpn1(*+*IRE)* mRNA (*P* < 0.05), and significantly higher levels of *Tfrc* and *Dmt1(*+*IRE)* mRNA (*P* < 0.05) compared to WT oligodendrocytes. However, no significant differences were reported in *L-ferritin* or *H-ferritin* expression between WT OPCs and WT oligodendrocytes.

## Discussion

The primary objective of our current study was to better understand the role of the multi-copper ferroxidases HEPH and CP in the CNS by studying their expression and the consequences of their loss in purified murine cortical astrocytes and oligodendrocytes.

We demonstrated that *Heph* was highly expressed in oligodendrocytes, while *Cp* was mainly expressed in astrocytes, and that the genes encoding both MCFs gene were only expressed at very low levels in primary cell cultures of OPCs. Iron efflux was impaired in *Cp* KO astrocytes and *Heph* KO oligodendrocytes, and this was associated with increased free radical production.

We found that loss of the *Cp* or *Heph* genes reduced *Fpn1* mRNA expression in astrocytes and oligodendrocytes respectively, with or without iron treatment. However, *Fpn1* expression was significantly increased after iron treatment in WT and *Heph* KO astrocytes or in WT and *Cp* KO oligodendrocytes. Those results suggest that MCFs are functional in brain cells and that CP and HEPH facilitate iron release from astrocytes and oligodendrocytes respectively in conjunction with FPN1. These findings are consistent with earlier studies on CP. The GPI-linked form of CP has been shown to be associated with FPN1 on the plasma membrane of astrocytes and is required for the export of iron from those cells *in vitro*^25,26^. It has also been reported that CP-mediated iron oxidation is necessary to release iron from FPN1. When GPI-CP (or another ferroxidase) is not present, FPN1 was internalized and rapidly degraded^27^.

*Tfrc* expression was significantly downregulated in *Cp* KO astrocytes following iron treatment, although no significant differences were found in WT or *Heph* KO astrocytes under the same conditions. However, *Tfrc* expression was markedly decreased in oligodendrocytes from each of the mouse lines studied following iron treatment. *Tfrc* expression can be affected by several factors, including iron levels and hypoxia^28^. The levels of *Tfrc* mRNA are elevated when the cellular iron content is low^29^, and decreased when cellular iron levels are high^24,30^. These changes are mediated by the well-described IRE-IRP system^31^. We also found that OPCs expressed *Tfrc* and that, as those cells matured, *Tfrc* gene expression was down. OPCs may require TFR1 to deliver iron to support their maturation and for maintaining myelination^24^. After myelination is complete and OPCs are mature, oligodendrocytes likely require less iron and TFR1 expression in oligodendrocytes is generally low^32,33^.

The expression of *Cp*, *Heph* and *Fpn1* was negligible in OPCs in our hands, contrasting with the relatively high expression of *Tfrc*, *Dmt1*, *H-ferritin* and *L–ferritin*. These results also support the concept that OPCs are more vulnerable to iron depletion as iron promotes their differentiation and myelination^24,34^.

Excessive free iron within cells is a potent driving force for oxidative stress as it catalyzes reactions that generate highly toxic hydroxyl radicals^35^. Thus, it is not surprising that levels of oxidative stress were significantly increased in both *Cp* KO astrocytes and *Heph* KO oligodendrocytes. It should be noted that the process of generating primary cultures of various cell types can, in itself, be associated with oxidative stress, but in our studies we did not observe overt oxidative stress in primary astrocyte or oligodendrocyte cultured from wild-type mice, suggesting this is unlikely to have a significant bearing on our findings. A previous study has demonstrated enhanced oxidant stress in cerebellar cells derived from neonatal *Cp* KO mice^36^, and our group reported lower activities of antioxidant enzymes in tissues of adult *Heph* and *Cp* KO mice *in vivo*^22^. In short, reduced iron efflux from key cell types in the CNS following the deletion of the MCFs is linked to enhanced oxidative stress and could be relevant to the development of neurological disorders such as Parkinson’s disease^37,38^, Alzheimer’s disease^39^, Rett syndrome^40^, and Huntington’s disease^41^.

In summary, our results cumulatively highlight the complex nature of impaired iron metabolism in astrocytes and oligodendrocytes following exposure to high iron. *Cp* is highly expressed in astrocytes while *Heph* is highly expressed in oligodendrocytes. These results are supported by our finding that *Cp* and *Heph* null mice demonstrate brain iron accumulation, oxidative stress, and altered expression of iron-related molecules in these distinct cell types. We conclude that CP (in astrocytes) and HEPH (in oligodendrocytes) may serve beneficial iron export and neuroprotective roles, thus providing further rationale for augmenting the activity of HEPH and CP to protect brain cells against damage in response to injury where iron overload has been implicated^9^.

## Materials and Methods

### Primary cell culture

Details of the *Heph* KO and *Cp* KO mouse strains have been previously described^23^. As both were on the C57BL/6 genetic background, C57BL/6 mice were used as the source of controlcells. All animal studies were carried out in accordance with the National Institutes of Health (NIH) guidelines and were approved by the Institutional Animal Care and Use Committee of Nanjing University.

Astrocytes were purified from the cerebral cortex of neonatal WT, *Heph* KO, and *Cp* KO mice as described previously^25,42^. Briefly, the cerebral cortex was triturated with a fire-polished pipette, passed through a 70 µm cell strainer, and centrifugated (400 × g for 5 min). Cells were then plated in high-glucose Dulbecco’s Modified Eagle’s Medium (DMEM) (Invitrogen) containing 10% fetal bovine serum (FBS, Invitrogen). Cells were maintained in 25 cm^2^ flasks (cells from the cerebral cortexes of 3 mice per flask) coated with poly-D-lysine at 37 °C in a 5% CO_2_ humidified incubator. After 10–12 days, confluent cultures were shaken overnight, and the floating cells were discarded. Cells were then cultured until the monolayers reached 80% confluence.

Since oligodendrocytes are less abundant in the brain than astrocytes, to obtain sufficient oligodendrocytes for study, we first isolated oligodendrocyte precursor cells (OPCs), then differentiated them *in vitro*. OPCs were enriched from the cerebral cortex of neonatal WT, *Heph* KO, and *Cp* KO mice as described previously^17,43^. Briefly, the cerebral cortex was triturated with a fire-polished pipette, passed through a 40 µm cell strainer, and plated onto 25 cm^2^ flasks (cells from 3 cerebral cortexes per flask) in neurosphere growth medium (NPM) consisting of DMEM/F12 with B27 supplement (Invitrogen) containing 20 ng/mL epidermal growth factor and 20 ng/mL bFGF. After 10 days, the NPM was progressively changed to oligosphere medium (NPM conditioned medium from B104 cells, 7:3) by replacing 25% of the original medium with the conditioned medium every other day. After 10 days in culture, the oligospheres were mechanically dissociated. The cell suspension was plated onto poly-D-lysine-coated 6-well plates for OPC proliferation in OPC medium consisting of Basal chemically defined medium (BDM: DMEM, 4 mM L-glutamine, 1 mM sodium pyruvate, 0.1%BSA, 50 mg/mL apo-transferrin, 5 mg/mL insulin, 30 nM sodium selenite, 10 nM D-biotin and 10 nM hydrocortisone) containing 5.0 ng/mL PDGF-AA and 5.0 ng/mL bFGF, or differentiation in oligodendrocyte differentiation medium (BDM containing 15 nM triiodothyronine, 10 ng/mL CNTF and 1x N-acetyl-L-cysteine)^17,43^. All reagents used in cell culture were from Sigma-Aldrich, unless otherwise specified.

### Immunofluorescent staining

To verify the identity and assess the purity of isolated glial cells, immunofluorescent microscopy was used. Cells plated on poly-D-lysine-coated coverslips were fixed in acetic acid/ethanol [5:95 (v/v)] at −20 °C for 10 min and then incubated with the relevant primary antibody for 30 min at room temperature, followed by three washes in Minimum Essential Medium (MEM) (Invitrogen) and subsequent incubation with secondary antibody for 30 min. Sections were mounted in a medium containing 4′,6′-diamidino-2-phenylindole dihydrochloride (DAPI) (100 ng/mL; Vector Laboratories) to show cell nuclei, and examined by confocal microscopy (Olympus FluoView FV10i; Olympus, Tokyo, Japan). The primary antibodies used were anti-GFAP (rabbit polyclonal antibody; 1:1000; Cat#ab7260, Abcam, MA) for astrocytes, anti-GalC (mouse monoclonal antibody; 1:100; Cat#MAB342, Merck Millipore) for oligodendrocytes, and anti-A2B5 (mouse monoclonal antibody; 1:100; Cat#MAB312, Merck Millipore) for OPCs. The secondary antibodies used were Dylight 488-conjugated goat anti-rabbit IgG (1:200; Cat#E032220-2, EarthOx) and Alexa Fluor 594-conjugated goat anti-mouse IgG (1:200; Cat#A-11005, Invitrogen).

### Iron efflux studies

Iron efflux from WT and *Cp* KO astrocytes was assessed as described previously^25^. Briefly, cultured astrocytes in 24-well plates were washed with serum-free DMEM twice and incubated in serum-free DMEM for 1 hour. Cells were then incubated in DMEM containing 40 µM FeCl_3_ and 1.8 mM L-ascorbate (molar ratio1:44) for 24 hours to load them with iron. The cells were then washed twice with serum-free DMEM and incubation was continued in the same medium. After 0, 12, or 24 hours, the cells were washed three times in phosphate-buffered saline (PBS). The concentration of non-heme iron in the cell pellets was measured as described below. The iron level within the cells at the 0 hour time point was set at 100%. Cellular iron efflux was quantified in triplicate in each experiment, and three separate experiments were conducted. All reagents used in the iron efflux studies were from Sigma-Aldrich.

Iron efflux from WT and *Heph* KO oligodendrocytes was assessed as described previously^17^. Briefly, cultured oligodendrocytes in 6-well plates were incubated with serum- and transferrin-free oligodendrocyte medium (SFM) for 1 hour to remove any transferrin-bound iron, then loaded with iron (as described above) for 6 hours. After iron loading, cells were washed twice with SFM, then fresh SFM was added to the cultures. At 0, 12, or 24 hours thereafter, cells were washed three times with PBS, and the non-heme iron content was measured as described below.

The concentration of non-heme iron in the primary astrocytes and oligodendrocytes was measured by a modification of the bathophenanthroline assay described by Torrance and Bothwell^44^. Briefly, 1 × 10^6^ cell pellets were put in a 1.5 mL acid-resistant tube and 1 ml of an acid solution (3 M HCl, 0.6 M trichloroacetic acid) was added. The samples and standards were then incubated for 20 hours in a 65 °C water bath. Then vortex the samples for 5 seconds each, spin down at maximum speed in a microfuge, and then cool down. Freshly prepared chromogen reagent (one volume 0.1% bathophenanthroline disulfonic acid with 1% w/v thioglycolic acid, five volumes saturated sodium acetate, and five volumes iron-free water) was added to wells in a clear plastic 96-well plate (200 μL/well). Sample or standard (4 μL per well) was added and mixed by pipet. The plate was incubated at room temperature for 30 minutes and the absorbance at 535 nm was measured in a microtiter plate reader. The concentration of iron in the samples was then calculated based on the constructed standard curve. Data are expressed as nmol Fe/10^6^ cells.

### Measurement of intracellular oxidative stress

Intracellular oxidative stress was assessed using the oxidation-sensitive fluorescent probe 2′,7′-dichlorodihydrofluorescein-diacetate (DCFH-DA) using a Reactive Oxygen Species Assay Kit (Cat#S0033, Beyotime Biotechnology). Cells were cultured on coverslips then loaded with iron for 24 hours as described above. After a further 24 hours in serum-free DMEM, the cells were incubated with 10 µM DCFH-DA at 37 °C for 20 min following the manufacturer’s instructions, then examined by confocal microscopy (Olympus FluoView™ FV10i; Olympus, Tokyo, Japan).

### Total RNA extraction and qRT-PCR analysis

Total RNA was isolated from primary cell cultures using TRIzol reagent (Invitrogen) according to the manufacturer’s instructions. The integrity of the RNA was assessed using denaturing (formaldehyde) agarose gel electrophoresis. Total RNA (3 μg) was reverse transcribed using a Transcriptor First stand cDNA Synthesis Kit (Roche Diagnostics USA) and quantitative real-time polymerase chain reaction (qRT-PCR) was used as previously described^23^ to measure the expression of the *Heph*, *Cp*, *Fpn1(*+*IRE)*, *L-ferritin*, *H-ferritin*, *Tfrc*, and *Dmt1(*+*IRE)* genes in different brain regions. All primers (Table 1) were designed using Primer3 software. Expression levels were normalized to those of the housekeeping gene *GAPDH*.

**Table 1:** Sequences of primers for Quantitative RT-PCR.

Targets	Forward primer	Reverse primer
*Cp*	TCTACCAAGGAGTAGCCAGGA	ATCTTCCCTCTCATCCGTGC
*Heph*	GAATTTTGCGAGCCGACCTT	TCATCCGCTTTCAGATACCC
*Fpn1(*+*IRE)*	TCGGTTCCTCTCACTCCTGT	GTGGAGAGAGAGTGGCCAAG
*Dmt1(*+*IRE)*	TAGGCTGTGCTCAAACCTACAGCA	TACATGAGAGCCAGGCATGGTAGA
*H-ferritin*	TAAAGAACTGGGTGACCACGTGAC	AAGTCAGCTTAGCTCTCATCACCG
*L-ferritin*	TGGCCATGGAGAAGAACCTGAATC	GCTTTCCAGGAAGTCACAGAGAT
*Tfrc*	GGTGTTGCGGCGAAGTCCAGT	ACTCAGTGGCACCAACAGCTCC
*GAPDH*	AACTTTGGCATTGTGGAAGG	GGATGCAGGGATGATGTTCT

### Statistical analysis

All values are presented as mean ± SEM. Unpaired Student’s t test was used to analyze gene expression and iron efflux data. Differences were considered statistically significant at *P* < 0.05. All statistical analysis was performed using GraphPad Prism 6 Software (GraphPad Software, San Diego, CA).
